# Integrated Transcriptomic and Metabolomic Analysis Reveals Myocardial Adaptation Mechanisms in Plateau Pikas (*Ochotona curzoniae*) at High Altitude

**DOI:** 10.3390/ani16152409

**Published:** 2026-08-05

**Authors:** Tian Luo, Xia Zhu, Yanrong Li, Xuefeng Cao, Zhenzhong Bai, Lan Ma, Shou Liu

**Affiliations:** 1 Medical College, Qinghai University, Xining 810001, China; 13108277021@163.com (T.L.); 18782369078@163.com (X.Z.);; 2 Research Center for High-Altitude Medical Sciences, Qinghai University, Xining 810001, China; 3 Qinghai–Utah Joint Research Key Lab for High-Altitude Medicine, Qinghai University, Xining 810001, China

**Keywords:** high altitude, hypoxic adaptation, *Ochotona curzonia*, transcriptomic analysis, metabolome analysis, evolution mechanisms

## Abstract

The Qinghai–Tibet Plateau, with an elevation of 4000 m above sea level, is rich in biological resources, and all the native creatures are exposed to extremely inhospitable environmental challenges. However, the detailed adaptation mechanisms of the native organisms to such an environment are still unclear. In this study, in-depth high-throughput RNA-Seq sequencing and metabolome data were deployed to depict the transcription and metabolism landscape of pikas living at 4630 and 2600 m altitude to elucidate the multilayer adaptation mechanisms of pikas to the environment of the Qinghai–Tibet Plateau. We identified significant variations in transcription and metabolism between pikas from 4630 and 2600 m. Various genes functioned in inflammatory-related pathways, including inflammatory mediator regulation of TRP channels and GnRH/HIF-1/p53/TNF signaling pathways, which were more active in pikas from 4630 m. Immune-response-related genes were overall downregulated in high-altitude pikas (e.g., genes for pro-inflammatory mediator synthesis and immune cell recruitment); this may represent a potential adaptive immunomodulatory mode in response to hypoxic stress. The expression of genes involved in cardiac muscle contraction and oxidative phosphorylation pathways was lower in pikas from 4630 m, leading to advantages for pikas in obtaining sufficient oxygen. Pikas from 4630 m have a more active metabolism relevant to amino acids and lipids, which provides energy for pikas to adapt to a high-altitude environment. Metabolome results reach a consensus with metabolic analysis from the transcriptome that pikas from 4630 m contain high levels of various amino acids and lipids. Overall, our study reveals multi-layer adaptive signatures in the left ventricle of plateau pikas at both transcriptional and metabolic levels, advancing our understanding of how *Ochotona curzoniae* adapts to high-altitude hypoxia on the Qinghai–Xizang Plateau.

## Simple Summary

The Qinghai–Tibet Plateau, over 4000 m high, has harsh conditions with little oxygen, yet it is home to unique animals like pikas. We still do not know exactly how these animals survive in such tough environments. To find out, we studied pikas living at two different heights—4630 m (very high) and 2600 m (lower)—using advanced scientific tools. We found that pikas at the higher altitude have special ways to adapt: they better protect their bodies from harm caused by a lack of oxygen, use oxygen more carefully by slowing down certain body processes, and make more energy from nutrients like proteins and fats. These findings help us understand how animals can thrive in extremely high-altitude locations.

## 1. Introduction

As the roof of the world, the Qinghai–Tibet Plateau is the highest and largest plateau on Earth, with an average elevation of more than 4500 m [1,2,3]. Native humans and animals in the Qinghai–Tibet Plateau are exposed to extremely inhospitable environmental challenges, including hypobaric hypoxia, extreme cold and arid conditions, and intense ultraviolet radiation [4,5]. Most research concerns how wildlife adapts to the environment at high altitude and how wildlife ensures sufficient oxygen intake and efficient delivery of oxygen to the tissues under long-term exposure to a hypoxic environment [6,7]. Indeed, native organisms of Tibet have evolved unique morphological, physiological, and genetic features to adapt to such a high-altitude, inhospitable environment [8]. Recently, some studies have been performed to elucidate the adaptation mechanisms of organisms in Tibet, including the yak [9], Tibetan antelope [10], groundtit [11], Tibetan Mastiff [12], Tibetan wild ass [13], and Tibetan dog [14]. However, due to the limitations of such species’ distributions, how high altitude can urge the evolution of native species to adapt to such an inhospitable environment remains unclear.

Unlike other animals, the plateau pika (*Ochotona curzoniae)* is broadly distributed in the Tibetan Plateau. As the main native mammal, the plateau pika has lived in the Tibetan Plateau for 20–30 million years [15] and evolved delicate adaptation mechanisms to the extreme hypoxia, cold, and intense ultraviolet radiation, preferring to live in 2500–5300 m mountain areas. The animal was recognized as a representative and typical animal of the plateau natives and is fully adapted to the inhospitable high-altitude environment [16]. It has also been reported in the literature that plateau pikas reduce gut microbial diversity and food fermentation ability to cope with the cold winter [17]. Since pikas can be used as a model organism for plateau animal research, elucidating the adaptation mechanism of the pika to the plateau environment will contribute to understanding the evolutionary adaptation mechanism of other native animals on the plateau. Recently, some studies have tried to explain the adaptation mechanisms of pikas to high altitude and deciphered the effects of hypoxia on the pulmonary circulation, liver, and neuroendocrine system [18]. For example, pikas from high altitude have a lower right ventricular weight than those living at sea level [19]. They can boost mitochondrial surface density, microvessel density, and myoglobin content in the heart to adapt to hypoxia exposure [20]. Meanwhile, pikas exhibited left ventricular hypertrophy, and the expression level of vascular endothelial growth factor was higher in pikas, leading to better cardiac efficiency to adapt to the severe hypoxic environment [21]. Despite this progress, the detailed adaptation mechanisms of pikas to high altitude remain unclear.

With the rapid development of multi-omics, large-scale data detection allows us to globally elucidate the adaptation mechanisms of animals to changes in the environment. As a representative next-generation technology, the transcriptome is a powerful tool for the identification of functional genes of organisms in different conditions. Integrating the metabolome and transcriptome could further decipher the adaptation mechanisms of animals to environmental changes. In this study, we focused on elucidating the global gene expression and metabolite variations between pikas from 4630 m and 2600 m altitude, respectively. Thus, comparative transcriptomics and metabolomics were performed on pikas from different altitudes. Our data show that there are significant variations in patterns of transcription and metabolites between pikas from 4630 m and 2600 m. Various signaling pathways and metabolic pathways were identified to be more active in pikas from 4630 m, which contribute to the adaptation of pikas to high-altitude hypoxia.

## 2. Materials and Methods

### 2.1. Sample Collection and Preparation

Pikas were captured in the Kekexili Reserve (4630 m a.s.l.; *n* = 6) and at the foot of the Laji Mountain (2600 m a.s.l.; *n* = 6) on the Qinghai–Tibet Plateau, Qinghai Province, China. All plateau pikas in this study were healthy adult males, 8–10 months of age and weighing 160 ± 10 g. Prior to sampling, all animals were maintained under stable and stress-free conditions, with no instances of fighting, vigorous exercise, or other interventions occurring within the 24 h before sampling. This was done to eliminate any potential confounding effects of strenuous activity on myocardial metabolism and gene expression. Pikas were anesthetized by intraperitoneal injection of 1% pentobarbital sodium (50 mg/kg body weight); anesthesia depth was judged by the disappearance of the corneal reflex and limb withdrawal reflex. After successful anesthesia, the animals were sacrificed by aortic exsanguination (rapid and complete bloodletting from the abdominal aorta) to minimize the stress response of the body and avoid the influence of stress hormones on myocardial tissue samples. The heart was rapidly dissected on ice. The intact free wall of the left ventricle was isolated and thoroughly rinsed with pre-cooled 0.9% sterile normal saline to wash out residual intracavitary blood for subsequent multi-omics analyses. All sampling was completed within 5 min. During the whole cardiac tissue dissection process, residual blood in the ventricular cavity was completely washed away with pre-cooled sterile 0.9% normal saline (4 °C). 

### 2.2. RNA Extraction

Total RNA was extracted from each sample using a mirVanaTM miRNA Isolation Kit (#AM1561, Ambion, Austin, TX, USA), following the manufacturer’s instructions. RNA purity was checked using the NanoPhotometer^®^ spectrophotometer (Implen CA, USA). An Agilent Bioanalyzer 2100 (Agilent Technologies, Santa Clara, CA, USA) was used to ensure RNA integrity by determining the RNA integrity number. Eligible total RNA was further purified using an RNeasy micro kit (Cat#74004, QIAGEN, GmbH) and RNase-Free DNase Set (Cat#79254, QIAGEN, GmbH, Hilden, Germany). Then, cDNA libraries for the 4630 m and 2600 m groups were constructed, and a Qubit 2.0 fluorometer (Thermo Fisher Scientific, Waltham, MA, USA) and Qubit dsDNA HS kit(Thermo Fisher Scientific, Waltham, MA, USA) were used to determine the library concentration. Finally, clusters were generated, and the sequencing reagent was prepared according to the HiSeq 2500 user guide using paired-end technology. We analyzed the data in real time using Real-Time Analysis (RTA) software (v1.17.21.3) (Illumina, Inc. San Diego, CA, USA) for Illumina sequencing, reference transcriptome assembly, ortholog identification, sequence alignment, expanded gene family annotation, divergence time estimation, phylogenetic tree reconstruction, molecular evolution analyses, and sequence availability.

### 2.3. Transcriptome Sequencing and Assembly

A total amount of 1.5 µg of RNA per sample was used as input material for the RNA sample preparations. Sequencing libraries were generated using NEBNext^®^ Ultra™ RNA Library Prep Kit for Illumina^®^ (NEB, Ipswich, MA, USA) following the manufacturer’s recommendations, and index codes were added to attribute sequences to each sample. Briefly, mRNA was purified from total RNA using poly-T oligo-attached magnetic beads. First-strand cDNA was synthesized using a random hexamer primer and M-MuLV Reverse Transcriptase. Second-strand cDNA synthesis was subsequently performed using DNA Polymerase I and RNase H. Remaining overhangs were converted into blunt ends via exonuclease/polymerase activities. After adenylation of the 3′ ends of DNA fragments, the NEBNext Adaptor with a hairpin loop structure was ligated to prepare for hybridization. In order to select cDNA fragments preferentially 250~300 bp in length, the library fragments were purified with the AMPure XP system (Beckman Coulter, Beverly, MA, USA). Then, 3 µL of USER Enzyme (NEB, Ipswich, MA, USA) was used with size-selected, adaptor-ligated cDNA at 37 °C for 15 min, followed by 5 min at 95 °C before PCR. Then, PCR was performed with Phusion High-Fidelity DNA polymerase, Universal PCR primers, and Index (X) Primer. Lastly, the PCR products were purified AMPure XP system (Beckman Coulter, Beverly, MA, USA), and library quality was assessed on the Agilent Bioanalyzer 2100 system (Agilent Technologies, Santa Clara, CA, USA).

Raw data (raw reads) in fastq format were first processed through in-house Perl scripts. In this step, clean data (clean reads) were obtained by removing reads containing adapters, reads containing poly-N, and low-quality reads from the raw data. At the same time, Q20, Q30, GC-content, and sequence duplication level of the clean data were calculated. All the downstream analyses were based on clean data of high quality.

The left files (read1 files) from all libraries/samples were pooled into one big left.fq file, and the right files (read2 files) into one big right.fq file. Transcriptome assembly was accomplished based on the left.fq and right.fq files using Trinity (v2.15.1) [22], with min_kmer_cov set to 2 by default and all other parameters set to default.

### 2.4. Differential Expression Analysis

Gene expression levels were estimated by RSEM (v1.3.3) [23] for each sample. Clean data were mapped back onto the assembled transcriptome, and read counts for each gene were obtained from the mapping results. Differential expression analysis of two conditions/groups was performed using the DESeq R package (1.10.1). DESeq provides statistical routines for determining differential expression in digital gene expression data using a model based on the negative binomial distribution. The resulting *p*-values were adjusted using Benjamini and Hochberg’s approach for controlling the false discovery rate. Genes with an adjusted *p*-value < 0.05 found by DESeq were assigned as differentially expressed. *p*-values were adjusted using the *q*-value [24]. *q*-value < 0.005 and |log_2_(Foldchange)| > 1 were set as the threshold for significant differential expression.

### 2.5. Gene Functional Annotation

Gene function was annotated based on the NCBI non-redundant protein sequences (Nr), KEGG Ortholog database (KO), and Gene Ontology (GO). Gene Ontology (GO) enrichment analysis of the differentially expressed genes (DEGs) was implemented by the GOseq R package based on the Wallenius non-central hypergeometric distribution [25], which can adjust for gene length bias in DEGs.

KEGG [26] is a database resource for understanding high-level functions and utilities of the biological system, such as the cell, the organism, and the ecosystem, from molecular-level information, especially large-scale molecular datasets generated by genome sequencing and other high-throughput experimental technologies (http://www.genome.jp/kegg/, accessed on 16 August 2024). We used KOBAS (v3.0) [27] software to test the statistical enrichment of differentially expressed genes in KEGG pathways.

### 2.6. Metabolites Extraction

A 50 mg myocardial tissue sample was transferred into a 2 mL tube, and 500 μL of pre-cooled extraction mixture (methanol/chloroform (*v*:*v*) = 3:1) with 10 μL of internal standard (adonitol, 0.5 mg/mL stock) was added. Samples were vortexed for 30 s and homogenized with a ball mill for 4 min at 35 Hz, followed by ultrasonication for 5 min in ice water. After centrifugation at 4 °C for 15 min at 12,000 rpm, 200 μL of supernatant was transferred to a fresh tube. To prepare the quality control (QC) sample, 40 μL of each sample was taken out and combined. After evaporation in a vacuum concentrator, 30 μL of methoxyamination hydrochloride (20 mg/mL in pyridine) was added and then incubated at 80 °C for 30 min, followed by derivatization with 40 μL of BSTFA reagent (1% TMCS, *v*/*v*) at 70 °C for 1.5 h. The samples were gradually cooled to room temperature, and 5 μL of FAMEs (in chloroform) was added to the QC sample. All samples were then analyzed by a gas chromatograph coupled with a time-of-flight mass spectrometer (GC-TOF-MS).

### 2.7. GC-TOF-MS Analysis and Data Preprocessing

GC-TOF-MS analysis was performed using an Agilent 7890 (Agilent Technologies, Santa Clara, CA, USA) gas chromatograph coupled with a time-of-flight mass spectrometer. The system utilized a DB-5MS capillary column. A 1 μL aliquot of the sample was injected in splitless mode. Helium was used as the carrier gas, the front inlet purge flow was 3 mL min^−1^, and the gas flow rate through the column was 1 mL min^−1^. The initial temperature was kept at 50 °C for 1 min, then raised to 310 °C at a rate of 10 °C min^−1^, and then kept for 8 min at 310 °C. The injection, transfer line, and ion source temperatures were 280, 280, and 250 °C, respectively. The energy was −70 eV in electron impact mode. The mass spectrometry data were acquired in full-scan mode with the *m*/*z* range of 50–500 at a rate of 12.5 spectra per second after a solvent delay of 6.25 min. 

Raw data analysis, including peak extraction, baseline adjustment, deconvolution, alignment, and integration, was finished with Chroma TOF (V 4.3x, LECO) software, and the LECO-Fiehn Rtx5 database was used for metabolite identification by matching the mass spectrum and retention index.

### 2.8. Data Preprocessing and Annotation

The raw data were processed with an in-house program developed using R and based on XCMS for peak detection, extraction, alignment, and integration. The in-house database was established with available authentic standards. Then, an in-house MS2 database (BiotreeDB) was applied in metabolite annotation. The cutoff for annotation was set at 0.3. Principal component analysis (PCA) and partial least squares discriminant analysis (PLSDA) were performed using Metaboanalyst 3.0. PCA, and PLSDA was performed on the normalized peak area of the metabolites in each sample. The annotated data were treated by mean centering and unit variance scaling. Therapy *p* < 0.05 and fold change > 2.0 were used to identify significantly different metabolites. The VIP values for metabolites were calculated using PLSDA.

## 3. Results

### 3.1. Transcriptomic Analysis of Left Ventricular Myocardial Tissues

#### 3.1.1. RNA-Seq Data Quality Control and Assembly

**Figure 1:**
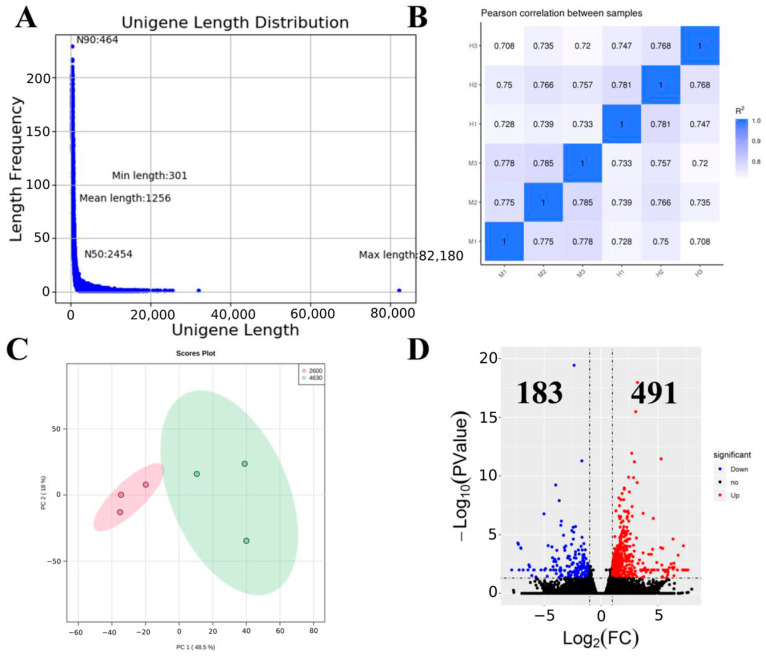
Overview of transcriptome profiles of pikas at 4630 m and 2600 m. (**A**) Sequence number distribution and cumulative length of contigs, transcripts, and unigenes (quality control analysis). (**B**) Unsupervised hierarchical clustering showing distinct transcription patterns between the two altitudes. (**C**) Principal component analysis (PCA) of transcriptomic profiles. the green ellipse covers samples from the 4630 m group, and the red ellipse covers samples from the 2600 m group (95% Hotelling’s T-squared confidence ellipse). (**D**) Volcano plots of differentially expressed genes (DEGs); upregulated DEGs (red) and downregulated DEGs (blue) (FDR < 0.05). Vertical dashed lines represent the threshold of |Log_2_(fold change)| = 1 for differential gene screening.

To investigate the adaptation mechanisms of the plateau pika to high-altitude hypoxia, comparative transcriptome analysis was performed on the left ventricular myocardial tissues of plateau pikas from 4630 m and 2600 m, respectively, with three biological replicates. M denotes the 2600 m group (sampled at the foot of Laji Mountain), and H denotes the 4630 m group (sampled in the Kekexili Reserve). RNA-seq generated high-quality sequencing data for six samples, with sufficient raw and clean reads obtained after filtering low-quality sequences and adapters (Table 1). These quality control metrics confirmed the reliability of the sequencing data for subsequent biological analysis. Furthermore, 86,302 unigenes were successfully assembled with a qualified length distribution and integrity (Figure 1A), and an average of 79.5% of reads were mapped to the reference genome of the plateau pika (*Ochotona curzoniae;* Table 1), verifying the specificity and validity of the assembled transcriptome for downstream differential expression analysis.

#### 3.1.2. Sample Clustering and Differential Expression Analysis

Subsequently, Principal Components Analysis (PCA) and unsupervised hierarchical clustering were used to investigate whether these variations are associated with high-altitude hypoxia (Figure 1B,C). The two score plots of the PCA models displayed a clear separation of samples from 2600 m and 4630 m altitude (Figure 1C), indicating that high-altitude hypoxia induced dramatic changes in the gene expression pattern of the plateau pika. The top two components explained 48.5% and 18.0% of the observed variance, represented by principal components (PCs) 1 and 2. Furthermore, unsupervised hierarchical clustering was performed, and a consensus was reached with the PCA results that all samples from 4630 m altitude were clustered together and clearly separated from the samples from the 2600 m group. These results suggest that the plateau pikas from 4600 m altitude possess specific mechanisms of high-altitude hypoxia adaptation at the transcription level.

Here, in our comparative transcriptome profiles, we identified 183 downregulated and 491 upregulated genes (FDR < 0.01) in the 4630 m vs. 2600 m comparison by at least a factor of 2 (FC > 2 or FC < −2). And these down- and upregulated genes were used for further functional analysis below to decipher the adaptation mechanisms of the plateau pika to high-altitude hypoxia.

#### 3.1.3. Functional Annotation of Differential Genes (GO and KEGG)

**Figure 2:**
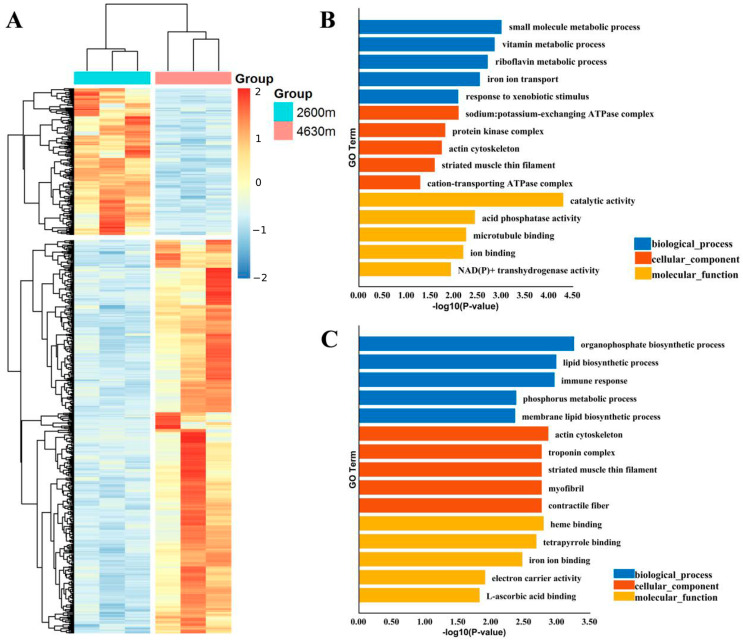
Function enrichment analysis on the significant differential genes in 4630 m vs. 2600 m. (**A**) Heatmap displaying the expression pattern of significant differential genes of pikas in the 4630 m vs. 2600 m comparison. Up- and downregulated genes are labeled in red and blue, respectively. The expression abundance is represented by the normalized FPKM values of the gene in each sample. (**B**,**C**) Gene Ontology enrichment analysis on up- (**B**) and downregulated (**C**) genes, respectively. Three main terms, molecular function, cellular component, and biological process, are shown in yellow, red, and blue, respectively.

Further gene function analysis was performed on these down- and upregulated genes. The expression patterns of these genes are shown in a heatmap. Functional enrichment analysis suggested that significantly downregulated genes (FDR < 0.05) were involved in 148 GO categories of three main groups, including biological process, molecular function, and cellular component (Figure 2C; Table S1). For biological processes, 82 categories were significantly enriched (*p* < 0.05), such as organophosphate biosynthetic process, lipid biosynthetic process, immune response, single-organism biosynthetic process, phosphorus metabolic process, membrane lipid biosynthetic process, and ion transmembrane transport (Figure 2C; Table S1). For categories relevant to cellular components in 4630 m vs. 2600 m, actin cytoskeleton, troponin complex, striated muscle thin filament, myofibril, sarcomere, and myofilament were significantly enriched (Figure 2C; Table S1). Among significant molecular function terms of downregulated genes from the 4630 m vs. 2600 m comparison, heme binding, tetrapyrrole binding, iron ion binding, aminoglycoside N-acetyltransferase activity, and electron carrier activity were the most significant terms (Figure 2C; Table S1). These data indicate that compared with the plateau pikas from 2600 m altitude, various biological processes of the plateau pikas from 4630 m altitude were reduced to a low level.

Subsequently, 491 upregulated genes between the 4630 m and 2600 m groups were annotated with GO terms (Figure 2B; Table S1). For GO term analysis on upregulated genes, genes were categorized into 187 functional groups (Figure 2B; Table S1), and the most represented five GO terms in each of the three main categories of biological process and cellular component were small-molecule metabolic process, vitamin metabolic process, riboflavin metabolic process, iron ion transport, response to xenobiotic stimulus, sodium:potassium-exchanging ATPase complex, protein kinase complex, actin cytoskeleton, striated muscle thin filament and cation-transporting ATPase complex, respectively. As expected, genes annotated with the GO terms relevant to respiratory action were found in the biological process category, including catalytic activity, acid phosphatase activity, microtubule binding, ion binding, and NAD(P)+ transhydrogenase activity. The results of our transcriptomic analysis thus provide authentic information suggesting that there are various advantages in the respiratory action of the plateau pika to adapt to the high-altitude 4630 m hypoxia stress, and they will also reduce consumption in other biological processes.

**Figure 3:**
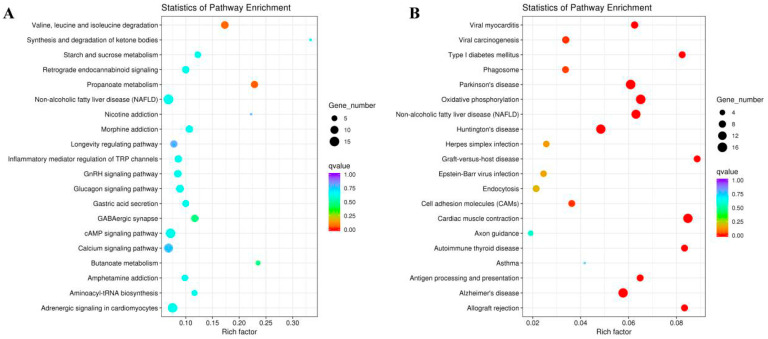
KEGG pathway enrichment analysis on differentially expressed genes from the 4630 m vs. 2600 m comparison. Pathway enrichment results of up- (**A**) and downregulated (**B**) genes in pikas from 4630 m. Each plot represents one pathway, and the color indicates the *p*-value of each pathway in the 4630 m vs. 2600 m comparison.

For functional analysis, 183 downregulated and 491 upregulated genes were identified in 69 and 231 KEGG functional pathways (Table S2; Figure 3). The pathway enrichment analysis on downregulated genes showed that the most represented were cardiac muscle contraction, oxidative phosphorylation, non-alcoholic fatty liver disease (NAFLD), cell adhesion molecules (CAMs), and endocytosis (Table S2; Figure 3B). These results imply that high-altitude hypoxia urged the plateau pikas from 4630 m to evolve a specific regulation of cardiac muscle contraction and oxidative phosphorylation to adapt to high-altitude hypoxia. 

To identify biological pathways active in the plateau pika in response to high-altitude hypoxia from 4630 m, further KEGG pathway enrichment analysis was performed on the upregulated genes against the KEGG database. The top 20 KEGG pathways identified from the 4630 m vs. 2600 m comparison are shown in Figure 3A (Table S2). In total, 17 pathways were significantly enriched based on 491 upregulated genes from the 4630 m vs. 2600 m comparison, including propanoate metabolism; valine, leucine and isoleucine degradation; GABAergic synapse; butanoate metabolism; morphine addiction; starch and sucrose metabolism; retrograde endocannabinoid signaling; adrenergic signaling in cardiomyocytes; the glucagon signaling pathway; gastric acid secretion; synthesis and degradation of ketone bodies; the cAMP signaling pathway; aminoacyl-tRNA biosynthesis; inflammatory mediator regulation of TRP channels; non-alcoholic fatty liver disease (NAFLD); the GnRH signaling pathway; and amphetamine addiction (Figure 3A). We noted that various metabolic pathways relevant to amino acids were active in the plateau pikas from 4630 m. Meanwhile, three signaling pathways were more active in the plateau pikas from 4630 m compared with those from 2600 m, including retrograde endocannabinoid signaling, adrenergic signaling in cardiomyocytes, and the cAMP signaling pathway (Figure 3A). These active signals could stimulate the left ventricular myocardial tissues of plateau pikas from 4630 m to ensure that plateau pikas can obtain adequate oxygen in response to high-altitude hypoxia stress. Additionally, variances in metabolic pathways reminded us that the specific composition of metabolites may also be involved in the adaptation mechanisms of the plateau pika to high-altitude hypoxia.

### 3.2. Metabolomic Analysis of Left Ventricular Myocardial Tissues

#### 3.2.1. Metabolite Profiling and Differential Metabolite Screening

**Figure 4:**
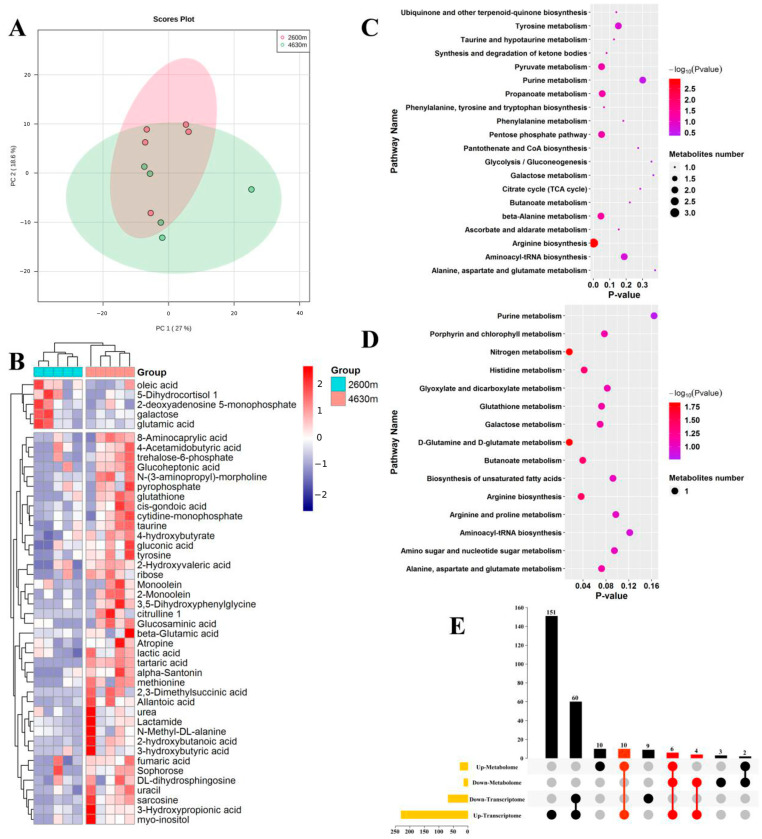
Integrated analysis of metabolome and transcriptome profiles of pikas from 4630 m and 2600 m. (**A**) Principal component analysis demonstrating the distinct biological variation among pikas from 4630 m and 2600 m. The green and red points represent the samples from 4630 m and 2600 m, respectively. All samples are within the 95% confidence interval (Hotelling’s T-squared ellipse) and represent experimental groupings. (**B**) Level of significantly differentially regulated metabolites in the 4630 m vs. 2600 m comparison. (**C**,**D**) Pathway enrichment analysis on the up- (**C**) and downregulated (**D**) metabolites in the 4630 m vs. 2600 m comparison. The scale and color represent the *p*-value of each pathway in this pairwise comparison. (**E**) Upset plots identifying the pathways that were commonly identified in the transcriptome and metabolome profiles.

To investigate the variations in metabolic patterns involved in adaptation mechanisms of the plateau pika to high-altitude hypoxia, five biological replicates of left ventricular myocardial tissues of plateau pikas from 4630 m and 2600 m were determined by metabolomics using a GC-MS platform. The PCA models on the two groups of metabolic profiles showed a clear separation between samples from 4630 m and 2600 m. Both PC 1 and PC 2 explained 45.6% of the variance in metabolic patterns among pikas from 4630 m and 2600 m (Figure 4A). The results suggest that the variations in metabolism are involved in the adaptation mechanisms of the plateau pika to high-altitude hypoxia. In total, through the threshold VIP > 1.0 and Log_2_FC > 1.0/< −1.0, we identified five metabolites that were downregulated in the 4630 m vs. 2600 m comparison, whereas the levels of 40 metabolites were higher in pikas from 4630 m than those from 2600 m (Table S3; Figure 4B). All downregulated metabolites and the top 10 upregulated metabolites are shown in Table 2, with their FAME values from the PLS-DA model. The relative abundance of each significantly differentially expressed metabolite is shown in Figure 4B. The results show that the contents of these compounds are consistent in each replicate from 4630 m and 2600 m, respectively, demonstrating their potential involvement in the adaptation mechanisms of pikas to high-altitude hypoxia.

#### 3.2.2. KEGG Pathway Enrichment of Differential Metabolites

To further investigate the function of these key hypoxia-related metabolites and the pathway that produces them, KEGG pathway enrichment analysis was performed on these down- and upregulated metabolites against the KEGG database (Figure 4). The results of downregulated metabolites showed that among 15 identified pathways, five pathways were significantly altered between pikas from 4630 m and 2600 m, including nitrogen metabolism, D-glutamine and D-glutamate metabolism, arginine biosynthesis, butanoate metabolism, and histidine metabolism (Figure 4D). Furthermore, pathway enrichment analysis on those 40 upregulated metabolites resulted in the identification of 28 biological processes, and four pathways were significantly more active in pikas from 4630 m, including arginine biosynthesis, beta-alanine metabolism, pyruvate metabolism, and the pentose phosphate pathway (Figure 4C). Similarly, this reaches a consensus with the results of the transcriptome results above that the variations are mainly identified from metabolism relevant to amino acids, such as alanine, aspartate, and glutamate metabolism; glutathione metabolism; and beta-alanine metabolism (Figure 4C).

### 3.3. Integrated Transcriptomic and Metabolomic Analysis

#### Identification of Common Hub Pathways

Considering the alterations in genes relevant to metabolism, we integrated the transcriptome and metabolome profiles to identify the hub pathways involved in adaptation mechanisms of pikas to high-altitude hypoxia. The results show that there are 20 pathways commonly identified in both the transcriptome and metabolome, such as butanoate metabolism; beta-alanine metabolism; propanoate metabolism; galactose metabolism; alanine, aspartate and glutamate metabolism; glutathione metabolism; and glycolysis/gluconeogenesis (Table 3). Thus, we propose that pikas can alter the metabolism relevant to energy and nutrients at both the metabolite and transcription levels to adapt to high-altitude hypoxia.

## 4. Discussion

This study presents an integrated transcriptomic and metabolomic analysis of left ventricular myocardial tissue from plateau pikas inhabiting two distinct altitudes on the Qinghai–Tibet Plateau (4630 m vs. 2600 m), with the objective of elucidating the adaptive mechanisms of this endemic species to high–altitude hypoxia. These analyses provide a multi-dimensional characterization of adaptive changes in the pika myocardium at both the transcriptional and metabolic levels. Principal component analysis revealed a clear separation in both transcriptomic and metabolomic profiles between high- and low-altitude populations, demonstrating that hypoxic stress drives systematic adaptive remodeling. The adaptive strategy of high-altitude pikas was found to operate through three primary mechanisms: (1) fine-tuned regulation of hypoxia-induced inflammatory responses; (2) optimization of myocardial energy balance via downregulation of oxygen-consuming pathways coupled with upregulation of alternative energy substrate metabolism; and (3) enhancement of antioxidant defense capacity to mitigate oxidative stress. The following discussion is structured around these three principal findings.

### 4.1. High-Altitude Pikas Adapt to Hypoxic Stress Through Fine-Tuned Regulation of Inflammatory Signaling Pathways

Hypoxic exposure typically induces an inflammatory response [28,29]; however, excessive or dysregulated inflammation can lead to tissue damage and fibrosis [30,31]. In the present study, multiple inflammation-related pathways were found to be activated in the myocardium of high-altitude pikas, yet this activation was accompanied by protective regulatory mechanisms countering potential injury. Transcriptomic analysis revealed that, relative to the 2600 m group, the myocardium of pikas from the 4630 m group exhibited significant enrichment of pathways including “inflammatory mediator regulation of *TRP* channels” (*p* = 0.012), the “TNF signaling pathway” (*p* = 0.031), and the “HIF-1 signaling pathway” (*p* = 0.043) (Figure 3A). This observation aligns with previous findings in other animal models, in which hypoxia has been demonstrated to activate *HIF-1α* and its downstream inflammatory cascade [32,33].

However, this inflammatory response in high-altitude pikas contrasts with typical pathological inflammation. Explosive upregulation of pro-inflammatory cytokines such as *IL-1β* and *TNF-α* was not observed. Rather, GO analysis revealed an overall downregulation trend in genes associated with the “immune response” (Figure 2C). This indicates that the inflammatory response in high-altitude pikas represents a controlled, adaptive process rather than an uncontrolled pathological state.

### 4.2. Remodeling of Myocardial Energy Metabolism: Reducing Oxygen-Dependent Energy Production and Enhancing Substrate Supply

The heart is a highly energy-demanding organ and is exquisitely sensitive to changes in oxygen supply [34]. A central finding of this study is that high-altitude pikas remodel myocardial energy metabolism through the coordinated downregulation of oxygen-consuming processes and enhancement of alternative energy substrate utilization.

KEGG enrichment analysis revealed significant downregulation of genes associated with the “cardiac muscle contraction” and “oxidative phosphorylation” pathways in the myocardium of high-altitude pikas (*p* < 0.05, Figure 3B). This finding represents an adaptive energy-conserving strategy employed by pikas under hypoxic conditions: reduced expression of cardiac-contraction-related proteins attenuates myocardial contractility, thereby decreasing oxygen consumption per unit time. Concurrently, downregulation of genes encoding mitochondrial electron transport chain complexes reduces the oxidative phosphorylation rate, thereby mitigating excessive reactive oxygen species (ROS) generation resulting from electron leakage under oxygen-limited conditions. This low-oxygen-consumption, high-efficiency strategy sustains basal physiological functions in pikas under hypoxic conditions.

Concurrent with the reduction in oxygen consumption, high-altitude pikas exhibited significant upregulation of amino acid and lipid metabolism. At the transcriptomic level, pathways including “valine, leucine and isoleucine degradation” (*p* < 0.001) and “propanoate metabolism” (*p* < 0.001) were significantly enriched (Figure 3A). The metabolomic data were in strong agreement with the transcriptomic findings, revealing significant elevations in multiple amino acids (e.g., leucine, isoleucine, and valine) and fatty acids (e.g., pentadecanoic acid and 2-monoolein) in the myocardium of the high-altitude group (*p* < 0.05, Table 2).

This phenomenon carries significant physiological implications: under hypoxic conditions, wherein glucose aerobic oxidation becomes increasingly constrained, the importance of fatty acids and amino acids as alternative energy substrates becomes increasingly pronounced [35,36]. Amino acids serve not only as direct substrates for the tricarboxylic acid (TCA) cycle to support energy production but also provide carbon skeletons for ketone body synthesis, thereby supplying additional fuel for the myocardium. The accumulation of fatty acids provides the myocardium with an efficient energy reserve. This metabolic shift—from glucose dependency toward amino acid and lipid utilization—constitutes a form of metabolic flexibility that is a hallmark adaptive characteristic of native high-altitude animals, ensuring adequate ATP supply to the heart even under conditions of limited oxygen availability [37].

### 4.3. Core Pathways of Metabolic Adaptation Revealed by Multi-Omics Integration: TCA Cycle Intermediates and Antioxidant Defense

Integration of transcriptomic and metabolomic data identified 20 common pathways exhibiting significant alterations at both molecular levels (Table 3). Among these, pathways related to glutathione metabolism and the TCA cycle merit particular attention.

Hypoxic conditions are prone to inducing reoxygenation injury and mitochondrial dysfunction, consequently leading to oxidative stress [38]. In this study, the glutathione metabolism pathway exhibited a trend toward differential regulation in the myocardium of high-altitude pikas (*p* = 0.072, approaching statistical significance). Notably, levels of key antioxidants and related metabolites—including precursors of reduced glutathione (GSH) and 2-hydroxybutyrate—were significantly elevated (*p* < 0.05). Consistent with these findings, transcriptomic analysis revealed upregulated expression of genes associated with electron transport chain components and NAD(P)+ transhydrogenase activity (Figure 2B). These coordinated multi-omics alterations indicate that high-altitude pikas are able to effectively scavenge hypoxia-induced ROS and protect cardiomyocytes from oxidative damage through enhanced GSH-mediated antioxidant capacity and improved electron transfer efficiency [39].

Although hypoxia is conventionally considered to suppress TCA cycle activity, significantly elevated levels of multiple TCA cycle intermediates—including fumarate and malate—were observed in the myocardium of high-altitude pikas. This observation aligns with the enrichment of pyruvate metabolism (*p* < 0.05) and butanoate metabolism (*p* < 0.05) pathways identified in the transcriptomic data. This seemingly paradoxical finding illuminates an additional dimension of metabolic adaptation: the accumulation of these intermediates may, on one hand, derive from enhanced anaplerosis (e.g., the conversion of glutamine to α-ketoglutarate); on the other hand, they may function as signaling molecules (e.g., fumarate) to activate downstream antioxidant or protective pathways (e.g., the Nrf2 pathway) [40]. Thus, the accumulation of TCA cycle intermediates represents not merely a reflection of energy metabolic status, but also an active cytoprotective strategy [41].

### 4.4. Limitations and Prospects

The sampling procedure of this study has certain limitations. Cardiac tissue retains metabolic activity while residual blood is washed away using pre-cooled normal saline. All rinsing steps were performed on ice. The processing time for each animal sample was standardized to minimize metabolic alterations. Even so, this washing process may introduce pre-analytical systematic bias during metabolomic analysis.

In addition, this study integrated transcriptomics and metabolomics and clarified the core hypoxic adaptive mechanisms of the left ventricle in plateau pikas. However, hypoxic adaptation in plateau pikas represents a systemic organismal response rather than a cardiac-restricted phenotype. Future work will expand our tissue sampling repertoire to encompass critical hypoxia-sensing organs including the lungs and brain, enabling multi-tissue integrated omics profiling to dissect organ-specific adaptive signatures and inter-organ crosstalk. Such multi-organ comparative analyses will offer a holistic, systemic framework to fully unravel the layered molecular strategies that underpin chronic high-altitude acclimatization in this endemic plateau mammal.

## 5. Conclusions

By integrating transcriptomic and metabolomic analyses of left ventricular myocardial tissues from plateau pikas (*Ochotona curzoniae*) at 4630 m and 2600 m altitude, this study identifies candidate molecular signatures linked to high-altitude hypoxic adaptation. Specifically, high-altitude pikas exhibit activation of hypoxia-responsive inflammatory pathways in the myocardium, accompanied by a downregulation trend in immune-response-related genes. This coordinated pattern orchestrates a controlled inflammatory response that mitigates potential hypoxia-induced tissue injury, enhancing oxygen utilization efficiency through downregulating the expression of genes involved in cardiac muscle contraction and oxidative phosphorylation pathways, and ensuring sufficient energy supply via augmented amino acid and lipid metabolism. Notably, integrated transcriptomic and metabolomic analyses jointly identified 20 pathways associated with hypoxia adaptation in plateau pikas, several of which exhibited consistent statistical significance across both datasets, such as butanoate metabolism and propanoate metabolism, indicative of coordinated transcriptional and metabolic regulation. These findings provide insights into multi-layered hypoxic adaptive characteristics in the left ventricle of plateau pikas, advance our understanding of evolutionary adaptation in native Qinghai–Tibet Plateau organisms facing extreme hypoxia, and offer a molecular reference for subsequent hypoxia-related research.

## Figures and Tables

**Table 1:** Characteristics of pikas’ transcriptome assembly.

Sample	Raw Reads	Clean Reads	Total Mapped
**M1**	44,286,824	43,500,024	80.20%
**M2**	41,125,626	40,222,276	79.62%
**M3**	50,423,222	49,553,150	79.28%
**H1**	52,239,862	51,520,092	79.90%
**H2**	50,737,066	49,899,270	79.28%
**H3**	44,802,096	44,137,214	78.82%
**Average**	47,269,116	46,472,004	79.52%
**Total**	283,614,696	279,000,000	-

Footnote: M = 2600 m low-altitude group; H = 4630 m high altitude group; *n* = 3 biological replicates. Raw reads: original sequencing data; clean reads: filtered high-quality reads; total mapped: mapping ratio to pika unigene library.

**Table 2:** List of significantly differentially expressed metabolites among pikas from 4630 m and 2600 m.

Name	4630 m	2600 m	LogFC	VIP
5-Dihydrocortisol	0.001577	0.004518	−1.51828	1.4559
Galactose	0.006418	0.01656	−1.36747	1.1351
2′-deoxyadenosine 5′-monophosphate	0.002369	0.005594	−1.23995	1.2933
Glutamic acid	0.365567	0.839329	−1.1991	1.1843
Oleic acid	0.024558	0.050023	−1.02638	1.2303
Tartaric acid	0.001637	9.1 × 10^−9^	17.45777	2.7085
Sarcosine	0.011772	0.001523	2.950014	2.2708
Allantoic acid	0.004099	0.000433	3.241708	2.1364
Alpha-santonin	0.000974	0.000241	2.016464	2.022
Tyrosine	0.054066	0.026342	1.037348	1.9925
Uracil	0.017399	0.007727	1.171078	1.9687
4-hydroxybutyrate	0.00468	0.001828	1.355875	1.936
2-hydroxybutanoic acid	0.029996	0.004765	2.654302	1.8984
Methionine	0.03469	0.007748	2.162546	1.8579
Ribose	0.0029	0.000812	1.83571	1.8513

Footnote: *n* = 5 biological replicates. FC = fold change (4630 m vs. 2600 m); VIP, variable importance in projection. Screening thresholds: VIP > 1.0, |log_2_FC| > 1, *p* < 0.05.

**Table 3:** List of pathway terms commonly identified in both transcriptome and metabolome profiles of pikas from the 4630 m vs. 2600 m comparison.

Pathway	*p*-Value		Regulation		Shared Altered Pathway
	Metabolome	Transcriptome	Metabolome	Transcriptome	
**Butanoate metabolism**	0.039288	0.008152	Increase	Increase	Significant
**Beta-alanine metabolism**	0.045823	0.284871	Increase	-	-
**Pyruvate metabolism**	0.049899	0.278245	Increase	-	-
**Pentose phosphate pathway**	0.049899	0.720958	Increase	-	-
**Propanoate metabolism**	0.049104	0.000227	Increase	Increase	Significant
**Galactose metabolism**	0.069875	0.52949	-	-	-
**Alanine, aspartate, and glutamate metabolism**	0.072391	0.098048	-	-	-
**Glutathione metabolism**	0.072391	0.608361	-	-	-
**Porphyrin and chlorophyll metabolism**	0.077407	0.697771	-	-	-
**Synthesis and degradation of ketone bodies**	0.080394	0.03652	-	Increase	-
**Glyoxylate and dicarboxylate metabolism**	0.082402	0.616011	-	-	-
**Biosynthesis of unsaturated fatty acids**	0.092332	0.385657	-	-	-
**Amino sugar and nucleotide sugar metabolism**	0.094802	0.364473	-	-	-
**Aminoacyl-tRNA biosynthesis**	0.12164	0.038652	-	Increase	-
**Purine metabolism**	0.16426	0.341127	-	-	-
**Citrate cycle (TCA cycle)**	0.28605	0.086189	-	-	-
**Glycolysis/gluconeogenesis**	0.35526	0.345846	-	-	-
**Phosphatidylinositol signaling system**	0.37687	0.349346	-	-	-
**Inositol phosphate metabolism**	0.39778	0.399413	-	-	-

Footnote: *p*-values from KEGG enrichment analysis. Increase = significantly higher level at 4630 m; “-” = no significant change. Shared significant pathways are enriched in both the transcriptome and metabolome datasets.

## Data Availability

The raw data (including RNA-seq sequencing reads and GC-TOF-MS metabolomic profiling data) and processed datasets (e.g., differentially expressed gene lists and KEGG/GO enrichment results) supporting the conclusions of this article are available from the corresponding author (Xuefeng Cao; E-mail: 2002980001@qhu.edu.cn) upon reasonable request.

## Supplementary Materials

The following supporting information can be downloaded at: https://www.mdpi.com/article/10.3390/ani16152409/s1. **Table S1**. Gene Ontology (GO) enrichment analysis of differentially expressed genes (DEGs) between the 4630 m and 2600 m groups. The table contains two sheets named “up” and “down”, corresponding to GO enrichment results of upregulated and downregulated DEGs, respectively. Each row records the GO accession, functional description, ontology type (biological process, cellular component or molecular function), raw over-represented p-value, corrected p-value, the count of enriched DEGs, total background genes and matched gene IDs for each GO term. **Table S2**. KEGG pathway enrichment results of DEGs from the two elevation groups. Two sheets are included, renamed as Upregulated and Downregulated (revised from original Chinese sheet names), which separately display enriched pathways of significantly increased and decreased genes, covering pathway ID, annotation, statistical values and enriched gene lists. **Table S3**. Quantitative data of all detected metabolites and screening results of differential metabolites in metabolomics. Three revised English sheets are provided: All metabolites contains quantification data of all 188 metabolites across biological replicates, together with VIP, log_2_FC and p-value indicators; Upregulated and Downregulated sheets summarize metabolites with significantly higher and lower contents in the 4630 m group compared with the 2600 m group.

## Abbreviations

The following abbreviations are used in this manuscript:

CAMs	Cell Adhesion Molecules
DEGs	Differentially Expressed Genes
FDR	False Discovery Rate
FC	Fold Change
GC-MS	Gas Chromatography–Mass Spectrometry
GC-TOF-MS	Gas Chromatography–Time-of-Flight Mass Spectrometry
GO	Gene Ontology
GnRH	Gonadotropin-Releasing Hormone
HIF-1	Hypoxia-Inducible Factor-1
KEGG	Kyoto Encyclopedia of Genes and Genomes
NAFLD	Non-Alcoholic Fatty Liver Disease
PCA	Principal Component Analysis
PLS-DA	Partial Least Squares Discriminant Analysis
RNA-Seq	RNA Sequencing
TNF	Tumor Necrosis Factor
TRP	Transient Receptor Potential
VIP	Variable Importance in Projection
